# Arjunolic acid reverses fluoxetine-induced alterations in testicular steroidogenic enzymes and membrane bound ionic pump imbalance through suppression of oxido-inflammatory stress and apoptosis

**DOI:** 10.5935/1518-0557.20230062

**Published:** 2024

**Authors:** Edozie Ojochem Lynda, Nwangwa Eze Kingsley, Oyovwi Mega Obukohwo, Ben-Azu Benneth, Emojevvwe Victor, Ovuakporaye I. Simon, Ejime Agbonifo-Chijiokwu, Onome B. Oghenetega

**Affiliations:** 1 Department of Human Physiology, Faculty of Basic Medical Science, Delta State University, Abraka, Delta State, Nigeria; 2 Department of Human Physiology, Adeleke University, Ede, Osun State, Nigeria; 3 Department of Pharmacology, Faculty of Basic Medical Science, Delta State University, Abraka, Delta State, Nigeria; 4 Department of Human Physiology, University of Medical Sciences, Ondo, Ondo State, Nigeria; 5 Department of Physiology, School of Basic Medical Science, Babcock University, Illisan-Romo, Ogun State, Nigeria

**Keywords:** fluoxetine, arjunolic acid, ATPase, steroidogenic enzymes, oxy-inflammation apoptosis

## Abstract

**Objective:**

The impact of the anti-depressant therapy on gonadal function has been recognized and discussed over the years. However, data to supplement our understanding of the impact of arjunolic acid (AA) therapies in protecting against FXT-induced gonadal dysfunction is lacking clear scientific evidence. Hence, this study aimed to investigate the possible effect of AA on fluoxetine-induced altered testicular function in rats.

**Methods:**

After 14 days acclimatization, Thirty-six (36) adult male rats were randomly divided into 6 groups (n=6). Rats in groups 1 received normal saline (10mL/kg); groups 2 & 3 were given AA (1.0mg/kg body weight) and AA (2.0mg/kg body weight), respectively; whereas, rats in group 4 were given FXT (10mg/kg/p.o/day), and groups 5 & 6 were given a combination of FXT (10mg/kg) + AA (1.0mg/kg body weight); and FXT (10mg/kg) + AA (2.0mg/kg body weight), respectively.

**Results:**

The results shows that FXT significantly altered testicular steroidogenic enzymes (3ß-HSD and 17ß-HSD) and proton pump ATPase (Na+/K+ ATPase, Ca_2_^+^ ATPase and H^+^ ATPase) activities, as well as testicular architecture when compared with controls. More so, FXT caused oxido-inflammation and apoptosis, as evidence by increases in MDA, MPO, TNF-α, IL-1ß, Caspase 3 and p53. However, AA at a different dose significantly ameliorated the destructive impacts of FXT on steroidogenic enzymes, proton pump ATPase as well as increased Bcl-2, SOD, CAT, GSH and improved testicular architecture in rats.

**Conclusions:**

AA reverses fluoxetine-induced alterations in testicular steroidogenic enzymes and membrane-bound ionic pump through suppression of oxido-inflammatory stress and apoptosis.

## INTRODUCTION

Depression is a common chronic recurrent mood disorder that influences both economic and social functions worldwide (Wille *et al*., 2008). Fluoxetine is one of the selective serotonin reuptake inhibitor drugs (SSRI) widely used in neurological disorder treatment such as depression, anxiety, bulimia nervosa, and obsessive-compulsive disorder (Guze & Gitlin, 1994; Jalili *et al*., 2014). Fluoxetine is known to be associated with some degree of sexual dysfunction in both men and women. In men, the most notable sexual side-effects may include impaired libido, erectile dysfunction and delayed ejaculation. The effect of fluoxetine (FLX) on male fertility and reproduction has been scientifically investigated. Long term or chronic administration of fluoxetine caused a decrease in spermatogenesis, levels of testosterone (T) and follicle stimulating hormone (FSH) and weights of reproductive organs in rats (Sakr *et al*., 2013; Bataineh & Daradka, 2007). Sexual disorders and decreased semen parameters were reported in patients treated with antidepressant fluoxetine (Agarwal *et al*., 2021). These sexual side effects can considerably affect a person’s lifestyle, and where this results in reduced compliance with medication, lead to less effective treatment of the primary psychiatric disorder (Göçmez *et al*., 2010). Moreso, fluoxetine is known to induce oxidative stress (Sakr *et al*., 2015), by overwhelming the cellular antioxidant systems, resulting in cellular damage.

Oxidative stress seen in reproductive disorders (Agarwal *et al*., 2003) has been linked to FXT-induced reproductive damage and germ cell apoptosis (Sakr *et al*., 2015; Soliman *et al*., 2017). Oxidative stress evoked by chronic FXT administration causes increased impaired testicular functions (Sakr *et al*., 2015). Severe oxidative challenges as seen in FXT toxicity overwhelms the body’s innate antioxidant mechanisms, thereby resulting in testicular oxidative injury. In this context, supplementation with antioxidant molecules such as arjunolic acid becomes imperative (Ghosh & Sil, 2013).

Arjunolic acid (AA: 2.3,23-trihydroxyolean-12-oic acid), found in nature as chiral triterpenoid saponin which is isolated from the bark of *T. arjuna*. This compound possess a variety of biological activities like, antiasthma agent (Kalola & Rajani, 2006), antitumor (Wille *et al*., 2009), wound healing (Chaudhari & Mengi, 2006), antifungal (Masoko *et al*., 2008), antibacterial (Djoukeng *et al*., 2005), and inhibition of insects growth (Bhakuni *et al*., 2002). Despite a variety of biological activities, AA is well-known for its cardioprotective role and proved to be beneficial against platelet aggregation and in lowering blood pressure, lipid level, myocardial necrosis, coagulation and heart rate (Ghosh & Sil, 2013). Its beneficial effects might be due to its potent antioxidant activity; which is demonstrated by its free radical scavenging activity. This compound has shown to be effective in eliminating radicals produced due to nitric oxide, superoxide and hydroxyl at a cellular level (Ghosh & Sil, 2013; Manna *et al*., 2007). Moreover, it possess protective effects toward cells and tissues against toxicity induced by drugs or heavy metals (Ghosh *et al*., 2010a; 2010b; Manna *et al*., 2007).

## MATERIALS AND METHODS

### Animals

We had thirty-six (36) Wistar rats weighing 150-250 g (6-8weeks old) in our experiment. The animals were kept in a controlled environment at about 25±2^o^C in 12:12 h day and night cycle. The animals were acclimatized for 14 days with unrestricted access to food and water. The study protocols used in handling the animals were in line with those established by the National institutes of Health (NIH) Guideline for the Care and Use of Laboratory Animals (Publication No. 85-23, revised).

### Drugs and chemicals

Fluoxetine (FXT) (Tesi Pharmaceuticals, Ughelli, Delta State, Nigerian) and arjunolic acid (AA) used in this study were bought from Sigma Aldrich, USA. FXT and AA were dissolved separately in 10 mL of saline immediately before use and administered orally. The doses and routes of Saline (Oyovwi *et al*., 2022), FXT at 10 mg/kg (Sakr *et al*., 2015) and AA at 1.0 and 2.0 mg/kg body weight were selected based on previous dose-response effects and preliminary investigation. More so, saline (10 mL/kg, p.o.) were administered as normal control and vehicle to naïve rats in different groups. The drugs were given between 8am and 9am and will be through oral route for 4 weeks.

### Experimental procedures

The rats were randomly divided into six groups-(n=6).

**Group I** This group served as control. The rats were treated with normal saline.

**Group II** The Rats in this group were treated with FXT alone at a dose of 10 mg/kg daily.

**Group III** This group was treated with *arjunolic acid* (at a dose of 1.0 mg/kg body weight) daily.

**Group IV** This group was treated with *arjunolic acid* (at a dose of 2.0 mg/kg body weight) daily.

**Group V** This group was co-treated with FXT (10 mg/kg) and arjunolic acid (1.0 mg/kg body weight) daily.

**Group VI** This group was co-treated with FXT (10 mg/kg) and arjunolic acid (2.0 mg/kg body weight).

### Sample collection and preparation

At the end of the experimental period, the animals were fasted overnight, after which the animal was slaughtered by cervical dislocation The testes were dissected out for biochemical assay (MDA, CAT, SOD, GPX, MPO, TNF-α, IL-1ß; 3ß-HSD, 17ß-HSD, Na+/K+ ATPase, Ca2+ ATPase, H^+^ ATPase) and histological studies. The reproductive organs were harvested, freed from adherent tissues and weighed on an electronic weighing. The testis was homogenized at 4^o^C in RIPA buffer containing 150 mM NaCl, 1 mM EDTA, 10 lg/ml PMSF, 1% Triton X-100 and 20 mM Tris-HCl, pH 7.4 in a glass Teflon homogenizer for 10s and centrifuged at 14,000x g for 20 min at 4^o^C. The supernatants were collected and immediately stored at -20^o^C until needed for biochemical assays.

### Oxidative status determination

#### *Estimation of* malondialdehyde (MDA) *in testicular cells*

This was done according to the method from Rice-Evans *et al*. (1996). The principle based on the reaction of Malondialdehyde, a product of lipid peroxidation with thiobarbituric acid to give a red species that was detected at 535 nm. The quantified MDA levels were expressed as nmole/mg protein.

#### Estimation of Superoxide dismutase (SOD) in testicular cells

This was estimated according to the method from (Misra & Fridovich, 1972). The principle is based on rapid autooxidation of adrenaline in aqueous solution to adrenochrome due to the presence of superoxide anions. The activity was determined with a spectrophotometer at 420 nm.

#### Estimation of catalase (CAT) in testicular cells

The concentration was determined with a spectrophotometer at 420 nm. This was determined according to the method from Aebi, 1984. Upon the addition of 30 mM H2O2 in 50 mM of phosphate buffer (pH 7.4) to sample, it is converted to oxygen and water. This action was stopped after three minutes by the addition of 1 mL of H2SO4 to the mixture, followed by 7.0 mL of KMnO4. Catalase (CAT) activity was estimated by a decrease in H2O2absorbance at 520 nm.

#### Estimation of glutathione redox status in testicular cells

Total-reduced glutathione (GSH) was determined by the method described by Jollow *et al.* (1974).

#### Determination of testicular proton pumps ATPase activities

Oyovwi *et al*. (2021)’s procedure was utilized to assay tissue homogenate to evaluate testicular Na+/K+ ATPase activity In a reaction mixture consisting of 0.8 mL of ice cold 10% (w/v) trichloroacetic acid (TCA), 1 mL of ammonium molybdate, and 1 mL of 9 percent ascorbic acid. A spectrophotometer was used to measure the absorbance at 725 nm. A reaction combination of 0.1 mL supernatant, 1mL 1.25 percent ammonium molybdate, and 1 mL 9 percent ascorbic acid was used to evaluate the activity of the testicular Ca2+ ATPase. The absorbance at 725 nm was then measured using a spectrophotometer. H+ activity was estimated based on a modified protocol described as previously published (Oyovwi *et al*., 2021).

#### Estimation of inflammation markers in testicular cells

Tumor necrosis factor-*α* (TNF-*α*) and interleukin-1*β* (IL-1*β*) levels were evaluated in the testicular homogenate using ELISA kits purchased from Thermo Fisher Scientific. The kits were used as per the manufacturer’s instructions. Meanwhile, testicular myeloperoxidase (MPO) activity was determined to quantify the buildup of polymorphonuclear leukocytes. This assay is based on hydrogen peroxide-dependent oxidation of guaiacol (Desser *et al*., 1972). Myeloperoxidase (MPO) assay was determined to Testicular neutrophil content .Activity of tissue MPO, an enzyme that is found predominantly in the azurophilic granules of polymorphonuclear leukocytes, correlates with the number of polymorphonuclear neutrophils determined histochemically in the inflamed tissues; it is therefore used as an indication of tissue neutrophil accumulation (Bradley *et al*., 1982). Testes MPO activity was measured using a procedure similar to that documented previously (Alturfan *et al*., 2012). The testes samples were homogenized in 50 mM potassium phosphate buffer ([PB], pH 6.0), and centrifuged at 41 400 g (10 minutes); pellets were suspended in 50 mM PB containing 0.5% hexadecyl trimethyl ammonium bromide (HETAB). After 3 freeze and thaw cycles, with sonication between cycles, the samples were centrifuged at 41 400g for 10 min. The aliquots (0.3 ml) were added to 2.3ml of reaction mixture containing 50mM PB, o-dianisidine, and 20mM H_2_O_2_ solution. One unit of enzyme activity was defined as the amount of the MPO present that caused a change in absorbance measured at 460 nm for 3 minutes. The MPO activity was expressed as U/mg tissue.

#### Estimation of apoptotic related protein markers in testicular cells

Testicular Caspase 3, p53, and Bcl-2 activities in testicular tissue homogenates were measured using caspase 3, p53, and Bcl-2 ELISA kits ( obtained from Sigma-Aldrich Chemical Co) as indicated by the manufacturer’s protocol.

### Histology of testes

The left testicle was harvested and immediately fixed in Bouin’s fluid for at least 5hrs. Each sample was dehydrated using ascending grades of alcohol. It was cleared with two changes of xylene, embedded in paraffin wax, trimmed, nicked and sectioned using a microtome and stained using hematoxylin and eosin (H&E) for the purpose of determining the general morphology.

### Statistical analysis

The data were analyzed using Graph pad prism 8 Biostatistics software (Graph pad Software, Inc., Lajolla, USA version 8.0). All the data were presented as mean ± SEM. Thereafter, the analysis was carried out by one way analysis of variance (ANOVA) and followed by post hoc test (Benferroni) for multiple comparisons. The level of significance for all the tests was set at *p*˂0.05.

## RESULTS

### Effects of arjunolic acid on flouxetine-mediated changes in oxidative status in rats’ testes

**Figure 1a-d:**
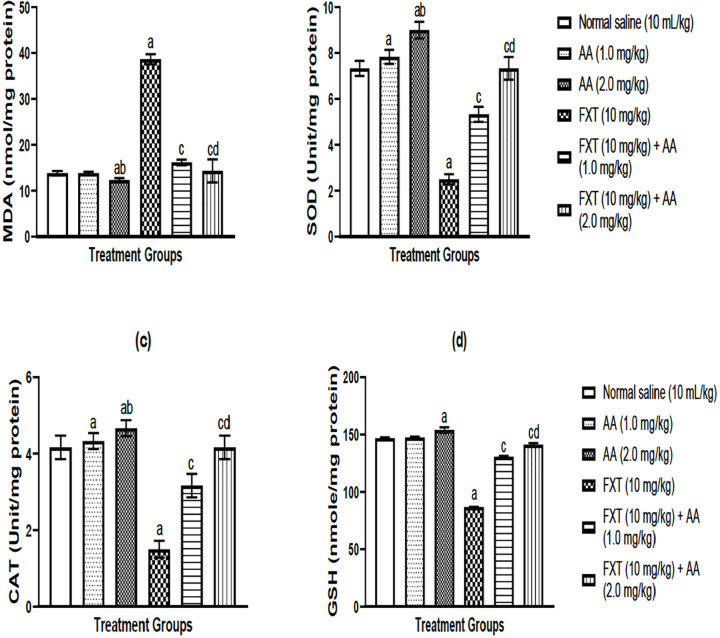
Arjunolic acid treatment abates fluoxetine-mediated oxidative stress in rat testes: MDA (a), superoxide dismutase, SOD (b), catalase, CAT (c) and glutathione peroxidase, GPx (d). The bars express mean ± S.E.M (n=6). One way ANOVA followed by *Bonferroni’s* post-hoc test revealed that there are significant differences between various treatment groups. ^a^*p*<0.05, as compared to control group; ^c^*p*<0.05, as compared to the FXT group; ^b^*p*<0.05, as compared to AA (1.0 mg/kg) group; ^d^*p*<0.05 when compared with FXT treated with AA (0.1 mg/kg/day p.o).

Fluoxetine treatment (10 mg/kg, p.o.) significantly (*p*<0.05) induced oxidative stress as evidenced by elevated MDA level (Fig. 1a), with a corresponding decrease in SOD (Fig. 1b), CAT (Fig. 1c) and GSH (Fig. 1d) levels were relative to controls. However, co-treatment with arjunolic acid different respective doses of 0.2 mg/kg, p.o. and 0.2 mg/kg, p.o. effectively (*p*<0.05) mitigates fluoxetine-induced oxidative stress by lowering MDA and increasing SOD, CAT, and GSH activities (Fig. 1a-d).

### Effects of arjunolic acid on fluoxetine-induced alteration in pro-inflammatory related markers in rats’ testes

**Figure 2a-b:**
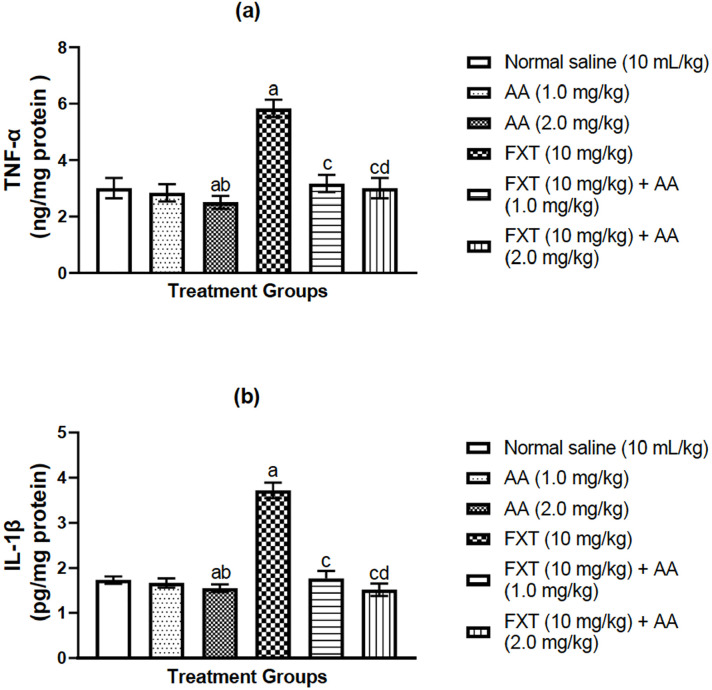
Arjunolic acid treatment abates fluoxetine-induced pro-inflammatory cytokines in rat testes: Tumor necrotic factor-,alpha TNF-α (a), and interleukin-1β (IL-1β) (b). The bars are represented as mean ± S.E.M (n=6). One way ANOVA followed by *Bonferroni’s* post-hoc test revealed that there are significant differences between various treatment groups. ^a^*p*<0.05, as compared to the control group; ^c^*p*<0.05, as compared to FXT group; ^b^*p*<0.05, as compared to the AA (1.0 mg/kg) group; ^d^*p*<0.05 when compared with the FXT-treated group with AA (0.1 mg/kg/day p.o.) group.

As presented in Fig. 2, one-way analysis of variance (ANOVA) following post-hoc test showed that fluoxetine treatment exhibited a significant increase in TNF-α (Fig. 2a) and IL-1β (Fig. 2b) levels when compared with control animals. Nevertheless, arjunolic acid co-treatment significantly (*p*<0.05) abates the increased pro-inflammatory cytokine levels as compared to fluoxetine-treated rats alone, data presented in Fig. 2a-b. More so, arjunolic acid administered at a higher dose of 2.0 mg/kg shows more significant effects as compared to the lower dose.

### Effects of arjunolic acid (AA) on fluoxetine (FXT)-induced apoptosis in rat testes

**Figure 3a-c:**
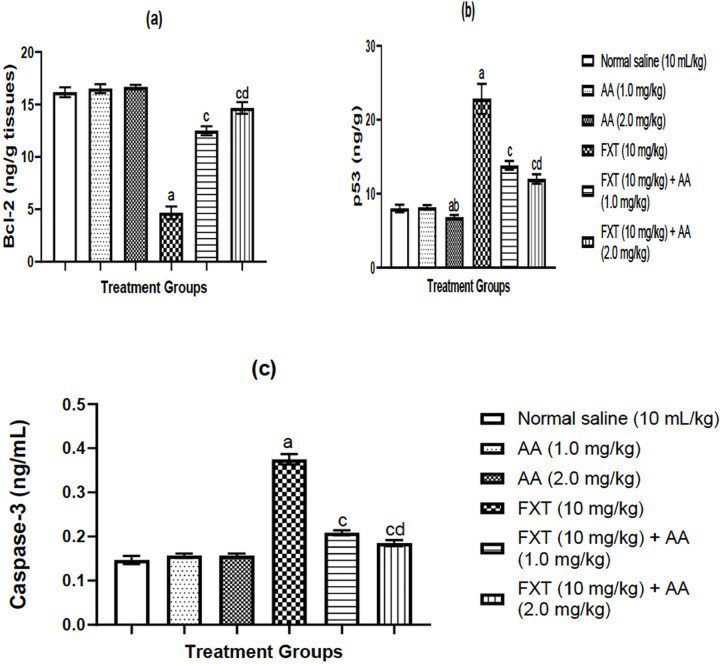
Arjunolic acid (AA) abates FXT-induced changes in apoptotic related protein in rats: BcL-2 (a) P53 (b), caspase-3 (c) in rat testes. The bars are represented as mean ± S.E.M (n=6) (One way ANOVA followed by Benferroni post hoc test). ^a^*p*<0.05, as compared to the control group; ^c^*p*<0.05, as compared to FXT group; ^b^*p*<0.05, as compared to AA (1.0 mg/kg) group; ^d^*p*<0.05 when compared with FXT treated with AA (1.0 mg/kg/day p.o) group.

FXT significantly (*p*<0.01) decreased Bcl-2 (Figure 3a) and increased in P53 (Figure 3b) and caspase-3 (Figure 3c) activities, respectively. However, this FXT-induced decrease in Bcl-2 and increase in p53 and caspases activities was reversed in rats co-treated with AA. BcL-2: B-cell lymphoma-2, P53: Tumor Protein-53.

### Effects of arjunolic acid on fluoxetine-induced increase in testicular neutrophil content in rats

**Figure 4:**
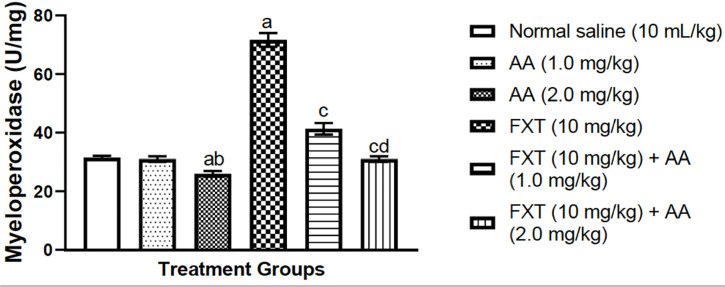
Arjunolic acid treatment mitigates fluoxetine-mediated increase in neutrophil content in rats’ testes. Bar is expressed as mean ± S.E.M (n=6) (One way ANOVA followed by Bonferroni’s post-hoc test). ^a^*p*<0.05, as compared to control group; ^c^*p*<0.05, as compared to FXT group; ^b^*p*<0.05, as compared to AA (1.0 mg/kg) group; ^d^*p*<0.05 when compared with FXT treated with AA (0.1 mg/kg/day p.o) group.

Fluoxetine (10 mg/kg, p.o.) invokes a significant (*p*<0.05) increase in testicular neutrophil content as indexed by a higher MPO level in rats (Figure 4). However, arjunolic acid co-treatment at 1.0 mg/kg and 2.0 mg/kg, respectively, significantly (*p*<0.05) mitigates this increase as compared to fluoxetine-treated rats alone. More so, arjunolic acid demonstrated a significant (*p*<0.05) decrease in testicular neutrophil as evidence by decreased in MPO as compared to the control group (Fig. 4).

### Effect of arjunolic acid (AA) on fluoxetine (FXT)-induced changes in steroidogenic enzymes activities in rat testes

**Figure 5a-b:**
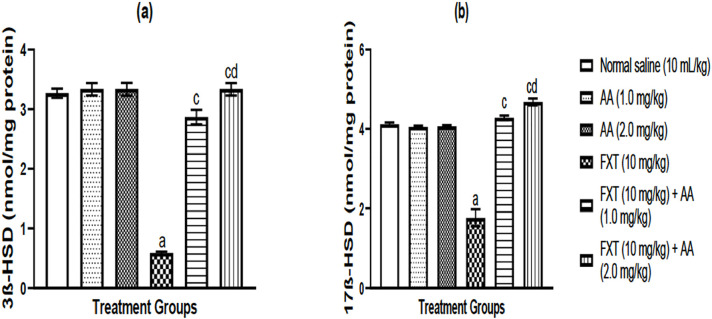
Arjunolic acid (AA) mitigates fluoxetine (FXT)-induced changes in testicular 3ßhydroxy steroid dehydrogenase (3ß-HSD) (a) and 17ß-steroid dehydrogenase (17ß-HSD) (b) activities in rats. Data are expressed as mean ± S.E.M. (n=6) (One-way ANOVA followed by Benferroni post hoc test). ^a^*p*<0.05, as compared to control group; ^c^*p*<0.05, as compared to FXT group; ^d^*p*<0.05 when compared with FXT treated with AA (1.0 mg/kg/day p.o) group.

Figure 5a-b presents the effect of arjunolic acid (AA) on fluoxetine (FXT)-induced changes in steroidogenic enzyme activities in rat testes. As indicated in Figure 5, FXT decreased 3ß-HSD (Figure 5a) and 17ß-HSD (Figure 5b) as compared to the control groups. AA attenuated the decline in 3ß-HSD (Figure 5a) and 17ß-HSD (Figure 5b) induced by FXT. Notably, treatment with AA alone did not produce any significant changes in 3ß-hydroxy steroid dehydrogenase (3ß-HSD) (Fig. 5a), and 17ß-teroid dehydrogenase (17ß-HSD) (Fig. 5b) levels when compared with normal control groups, respectively.

### Effect of arjunolic acid (AA) on fluoxetine (FXT)-induced alteration in membrane bound Ionic pump activities in rat testes

**Figure 6a-c:**
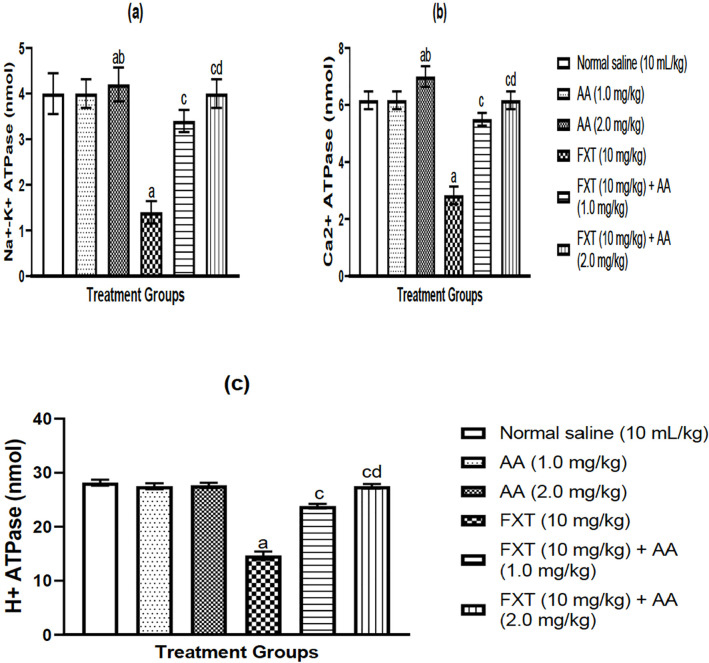
Arjunolic acid (AA) increases ionic-pumps activities in rat testes submitted to FXT: Na^+^/K^+^ ATPase (a), Ca^2+^ ATPase (b), H^+^ ATPase (c) activities. The bars are represented as mean ± S.E.M (n = 6) (One way ANOVA followed by Bonferronins post-hoc test). ^a^*p*<0.05, as compared to control group; ^c^*p*<0.05, as compared to FXT group; ^b^*p*<0.05, as compared to AA (1.0 mg/kg) group; dp<0.05 when compared with FXT treated with AA (1.0 mg/kg/day p.o) group.

In FXT-treated rats as shown in Fig. 6a-c, one-way ANOVA followed by post-hoc test revealed a significant decrease in Na^+^/K^+^ ATPase (Fig. 6a), Ca^2+^ ATPase (Fig. 6b) and H^+^ ATPase (Fig. 6c) activities as compared to normal control groups. In comparison to FXT-treated rats, AA treatment dramatically corrected FXT-mediated Na+/K+, Ca2+ Mg2+ ATPase changes (Fig. 6a-c).

### Effects of arjunolic acid on fluoxetine-induced histopathological changes in the testes of rats

**Figure 7a-f:**
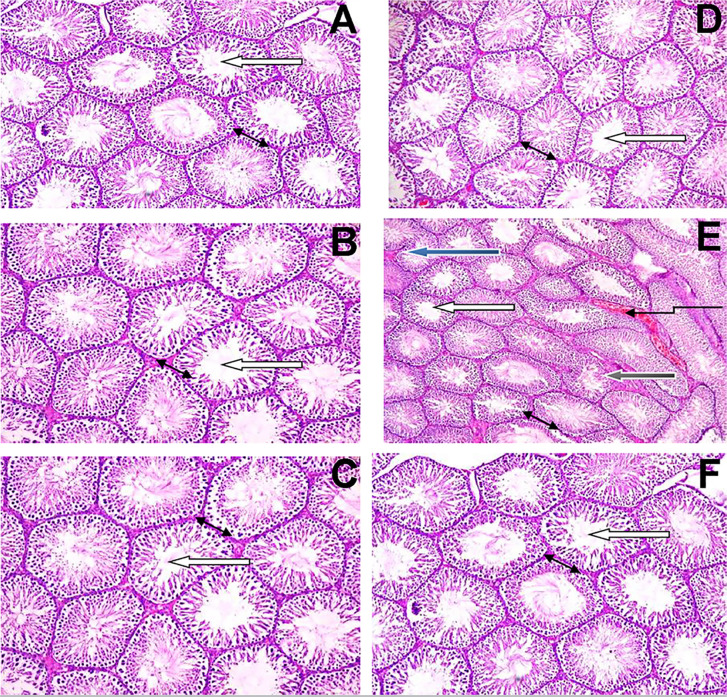
Photomicrographs showing the reversal effects of quercetin therapy on endosulfanmediated histopathological changes in rats testes. A: Control (10 mL/kg of saline); B: AA (1.0 mg/kg); C: AA (2.0 mg/kg); D: FXT (10 mg/kg); E: FXT (10 mg/kg) + AA (1.0 mg/kg); E: FXT (10 mg/kg) + AA (2.0 mg/kg). For plate 1, slides A, B and C revealed normal testicular architecture with seminiferous tubules, normal matured sperm stage. Slide D revealed some pyknotic cell, sloughing/degenerated germ cell layer and less spermatozoa within their lumen and also shows vascular congestion. Different atrophic seminiferous tubules, thickened pyknotic propria enveloping the tubular cells, degenerated and necrotic germ cells of the seminiferous tubules. Slide E and F are associated with showing normal testicular architecture Blue arrow- few seminiferous tubules showing maturation arrest at secondary level; Slender arrow- represent vascular congestion and pinkish homogenous mass; Black-degenerated germ cell layer; White arrow- seminiferous tubule with normal sperm maturation; Spanned arrow- The interstitial spaces show normal Leydig cells. H & E (Hematoxylin-eosin) stain: Original magnification x400, Calibration bar = 0.01 mm (10 µm) for the entire plate.

The effects of arjunolic acid on fluoxetine-invokes histopathological changes in the rats’ testes is presented in Figure 7a-f. Arjunolic acid alone produced no changes on the testicular architecture, seminiferous tubules and spermatozoa (spermatogenesis) when compared to the normal control group. In this study, the rats treated with fluoxetine (10 mg/kg/day) had a reduced spermatogenesis based on low spermatocytes in most of the seminiferous tubules, degenerated seminiferous tubules and necrosis with atrophy, dead pyknotic cells, homogenous and vascular congestion when compared with normal controls, which were repaired by arjunolic acid at different administered doses (1.0 mg/kg and 2.0 mg/kg/day).

## DISCUSSION

This study established the therapeutic efficacy of arjunolic acid (AA) to attenuate fluoxetine-induced alterations in testicular steroidogenic enzymes and membrane-bound ionic pump imbalance through the suppression of oxido-inflammatory stress and apoptosis in male rats. The AA has been well documented by numerous investigators not to be effective in eliminating radicals produced due to nitric oxide, superoxide and hydroxyl at the cellular level (Ghosh & Sil, 2013; Manna *et al*., 2007) and as a result, it is likely to be safe for human consumption in terms of reducing a variety of debilitating health effects. FXT have long been known to cause oxidative stress related to reproductive damage and germ cell apoptosis (Agarwal *et al*., 2003; Sakr *et al*., 2015; Soliman *et al*., 2017). More so, the therapeutic implication of AA supplementation on human and animal health has been widely documented (Wille *et al*., 2008; 2009; Chaudhari & Mengi, 2006; Kalola & Rajani, 2006; Masoko *et al*., 2008; Djoukeng *et al*., 2005; Ghosh & Sil, 2013; Ghosh *et al*., 2010a; 2010b; Manna *et al*., 2007) however, it appears that this is the first study to look into the effects of AA on fluoxetine-induced alterations in testicular steroidogenic enzymes and membrane-bound ionic pump imbalance.

Numerous investigations have presented the regulatory androgenic function and oxidative phosphorylation role of testicular steroidogenic enzymes (3β-HSD and 17β-HSD) in the testes (Dey *et al*., 2019; Oyovwi *et al.,* 2021; 2022). Specifically, 3β-HSD and 17β-HSD are known to convert androstenedione into testosterone in the seminiferous tubes of testes, which strengthens sexual performance and gamete fertility (Gray *et al*., 2018). In this study, FXT was found to cause significant decreases in testicular 3β-HSD and 17β-HSD levels, which are indicative of reduced levels of testosterone, reduced sperm maturation, low acrosomal reaction and fertilization. The protective effects of AA against FXT-induced biogenic depletion of 3β-HSD and 17β-HSD; therefore, suggesting improved testicular function and sperm maturation as well as reproductive enhancing properties (Oyovwi *et al*., 2021).

The membrane surface is responsible for ionic pump ATPase homeostasis and permeability. Hence, modifications of the surface by lipid peroxidation alters the Thiol (SH)-containing Ion-activated adenosine triphosphatases (ATPases). It is important to note that ionic pumps such as Na^+^, K^+^, and Ca^2+^, play a critical role in the exchange of metabolites between Sertoli and developing germ cells; and they are markers of the metabolic state of germinal epithelium, i.e spermatogenesis and testicular metabolism (McDermott *et al*., 2015). Most specifically, H+-ATPase-rich cells are involved in the acidification of the epididymis and vas deferens segments, a biological mechanism that is critical for sperm maturation and storage (Visconti, 2009). Impaired acidification of these segments has been reported to slow down sperm maturation, in part, due to ATPase disruption (Oyovwi *et al*., 2021). In our study, FXT therapy caused decreased testicular Na+K+-, Ca2+- and H+-ATPase activities, suggesting altered testicular ionic pump ATPase balance. But notwithstanding, treatments with *AA significantly mitigated against the effects caused by FXT on* Na^+^/K^+^ ATPase, Ca^2+^ ATPase and H^+^ ATPase activities, which may suggest increased membrane function and enhanced sperm motility cellular constituents; thus leading to improved spermatogenesis and sperm function.

Oxidative stress is one of the major contributors in the mechanism underlying testicular damage induced by FXT (Sakr *et al*., 2015; Soliman *et al*., 2017; Beeder & Samplaski, 2020) Lipid peroxidase (MDA), a known indicator of oxidative injury, which has been demonstrated to play a crucial role in cytotoxicity and cellular dysfunction due to its ability in cell membrane disruption (Gutteridge, 1995). Our results show FXT-induced testicular oxidative damage, as evidenced by elevated testicular MDA levels; also caused significant depletion in GSH, SOD and catalase activity in rats. This elevated value in MDA confirmed the ability of FXT to disturb the hemostasis in the oxidative status in testicular tissue. Co-administration of AA to FXT treated rats demonstrated a decreased testicular MDA level when compared to the FXT-treatment alone; moreover, increased the antioxidants parameters represented in GSH, SOD and CAT, these results evidenced the anti-oxidative effect of AA against FXT-induced oxidative stress in testicular tissues (Sakr *et al*., 2015; Soliman *et al*., 2017; Beeder & Samplaski, 2020).

Besides the direct adverse effects on testicular tissues, free radicals may seem to also trigger the buildup of leukocytes in the testicular tissue, and this may cause tissue injury indirectly through activated neutrophils. Activated neutrophils are known to induce tissue injury through the production and release of reactive oxygen metabolites and cytotoxic proteins (e.g. proteases, myeloperoxidase, lactoferrin) into the extracellular fluid. When neutrophils are stimulated by various stimulants, myeloperoxidase (MPO), as well as other tissue-damaging substances like nitrite, peroxynitrite and protein carbonyl are released from the cells as an index of neutrophil infiltration. Neutrophil infiltration mediated ROS is most often accompanied by inflammation and apoptosis (Chatterjee, 2016; Nna *et al*., 2017; Kannan & Jain, 2000). And since the neutrophil infiltration mediated ROS is an important event for the acute inflammation, increase in MPO activity due to FXT exposures may cause inflammation and damage the testicular organs (Soliman *et al*., 2017). Nevertheless, FXT co-treatment with AA attenuated the increase in the level of oxidant enzymes activities (myeloperoxidase) of testes as compared to FXT-treated rats alone. This present study is in line with the findings of Sumitra *et al*. (2001), Hemalatha *et al*. (2010), Miriyala *et al*. (2015), Dawé *et al*. (2018), which state that AA treatment protected testes against chemotoxicants.

Growing evidence advocates that apoptosis plays a central role in the pathogenesis of FXT-induced male testicular toxicity (Khaksar *et al.,* 2017). Actually, the protein bcl-2 is actively involved in apoptotic pathways. Accordingly, mis expression of bcl-2 up-regulates the permeability of mitochondrial membrane, which in turn results in intensive release of cytochrome C from mitochondrial inner-membrane space into the cytoplasm (Marsden *et al*., 2002). Moreover, sequestration of pro-caspases and inhibiting self-cleavage of caspases are known as another possible mechanism for anti-apoptotic activity of bcl-2 (Marsden *et al*., 2002; Youle & Strasser, 2008). Thus, we can hypothesize that, decreased expression of the bcl-2 may trigger the apoptosis pathway partially by up-regulating permeability of mitochondrial membrane and/or by enhancing self-cleavage of caspases. In fact, the decrease observed in protein level of Bcl-2 activities could be directly affected by the p53 expression as a part of transcription-independent programed cell death (Erster & Moll, 2005). Actually, the cytosolic p53 binds to pro-apoptotic (Bax and Bak) bcl-2 family proteins, which in turn leads to enhancing the permeability of mitochondrial membrane (Petros *et al*., 2004; Talos *et al*., 2005; Oyovwi *et al*., 2023). Thus, overexpressed p53, itself, acts as antagonist of anti-apoptotic (bcl-2 and bcl-xL) bcl-2 proteins (Kermer *et al*., 1999; Soleimani *et al*., 2012). More importantly, our data showed that, FXT triggered apoptosis, as evidenced by decreased Bcl-2 levels, which could be suggested through the up-regulation of P53 and caspase-3 pathway, reported in this study. Thus, we can come close to this fact that, the FXT-inducing increased in the p53 protein expression promotes the apoptotic pathway in spermatogenesis cell lineages. In line with this issue, the p53 is a sequence-specific transcription factor, which is activated by diverse forms of cellular stress and it is known to mediate the cycle arrest in response to cellular stress (Puzio-Kuter, 2011; Li *et al*., 2012). Caspase-3 overexpression might induce Leydig cell death (Morgan *et al*., 2015). Leydig cells play a key role in testosterone production; therefore, effecting Sertoli cell function (Shima *et al*., 2013). Serum testosterone levels show a significantly decreased concentration in FXT treated rats, which could be characterized by Leydig cells atrophy and germ cell degeneration. Depletion in testosterone concentration has been implicated to be associated with spermatogenesis impairment. In contrast, AA co-treatment induced a significant decrease in caspase-3 concentration, P53 level in FXT+AA group which reflects the anti-apoptotic effect of AA in testicular apoptosis induced by AA. In accordance with our finding, we reported the anti-apoptotic effect of AA against FXT- induced germ cell apoptosis (Manna *et al*., 2008; Al-Gayyar *et al*., 2014). AA co-administration with FXT induced up-regulation in Bcl-2 by a significant value in respect to FXT treatment alone, this result explains the ability of AA to act via P53 and caspase-3 pathway, thus inhibiting apoptosis in order to abrogate the cytotoxic impacts of FXT on testicular tissue.

FXT treatment induced a significant elevation in TNF-α and IL-1β level in FXT group, which proved the inflammatory effect of FXT on testicular tissues (Soliman *et al*., 2017). TNF-α, as well as IL-1β are pro-inflammatory cytokine that regulates multiple cellular processes in testes (Cheng & Mruk, 2002; Tesi *et al*., 2022). TNF-α increment could induce spermatogenesis impairment by decreasing sperm viability and increased sperm abnormalities (Ramonda *et al*., 2014). FXT co-administered with AA showed significant depletion in TNF-α and IL-1β in FXT+AA group, in comparison with FXT treated rats only. This result is in accordance with previous findings, which suggests the anti-inflammatory activity of AA on testicular inflammation induced by FXT (Manna *et al*., 2008; Al-Gayyar *et al*., 2014). The mechanism of protective efficacy of AA as an antioxidant and anti-inflammatory properties could be explained to be due to their ability to antagonize the activity of arachidonic acid; thereby reducing the production of inflammatory and chemotactic derivatives and suppressing cell-mediated immune responses.

Our histopathological findings on testicular architecture in the present study revealed degenerated seminiferous tubules and necrosis with the presence of atrophy, dead pyknotic cells, homogenous and vascular congestion, spermatogenesis disruption; thus suggesting the involvement of intrinsic mechanisms in the action of FXT on the germ cells, probably due to its ability to pass through the BTB. Interestingly, all these deleterious effects of FXT-induced testicular damage were ameliorated by the administration of AA. The fact that treatment with taurine+NAC was able to reverse the effects of FXT on testicular architecture, indicating a possible restorative efficacy of these compounds.

## CONCLUSION

In conclusion, this study appears to demonstrate that AA protects against testicular damaged caused by FXT. This gonad-protective effect of AA seems to be closely implicated with the restoration of testicular steroidogenic enzymes and activation of membrane bound proton pump ATPase activities, thus mediating inhibition of apoptosis and oxy-inflammation. Altogether, our results suggest that testicular steroidogenic enzymes and proton pump ATPase play a key role in testicular and sperm membrane function necessary for spermatogenesis. As a result, AA may be a new treatment option for drug-toxicant-related male reproductive impairment.

## Abbreviations

3ß-HSD: 3ß-hydroxysteroid dehydrogenase 17ß-hydroxysteroid dehydrogenase
17ß-HSD: 17ß-hydroxysteroid dehydrogenase
MDA: malonaldehyde
SOD: superoxide dismutase,
CAT: catalase (CAT),
GSH: reduced glutathione,
MPO: myeloperoxidase,
TNF-α: tumor necrosis alpha
IL-1β: interleukin-1beta
Na^+^/K^+^ ATPase: Sodium potassium adenosine triphosphate
Ca^2+^ ATPase: Calcium ion adenosine triphosphate
H^+^ ATPase: Hydrogen ion adenosine triphosphate
PUFAs: Polyunsaturated fatty acids
